# Isoflavone and Protein Constituents of Lactic Acid-Fermented Soy Milk Combine to Prevent Dyslipidemia in Rats Fed a High Cholesterol Diet

**DOI:** 10.3390/nu6125704

**Published:** 2014-12-10

**Authors:** Maki Kobayashi, Shintaro Egusa, Mitsuru Fukuda

**Affiliations:** 1 Department of Nutritional Management, School of Health Sciences, Hyogo University, 2301 Hiraoka-cho shinzaike, Kakogawa, Hyogo 675-0195, Japan; E-Mail: makik@hyogo-dai.ac.jp; 2 Research and Development Division, MARUSAN-AI Co., Ltd., 1 Aza-Arashita, Nikki-cho, Okazaki, Aichi 444-2193, Japan; E-Mail: shintaro.egusa@marusanai.co.jp; 3 Department of Food Science and Nutrition, School of Human Environmental Sciences, Mukogawa Women’s University, 6-46 Ikebiraki-cho, Nishinomiya, Hyogo 663-8558, Japan

**Keywords:** isoflavone, soy protein, fermented soy milk, cholesterol, lipid metabolism

## Abstract

A high cholesterol diet induces dyslipidemia. This study investigated whether isoflavone aglycones in lactic acid-fermented soy milk (LFS) improve lipid metabolism in rats fed a high cholesterol diet. Male Sprague-Dawley rats aged seven weeks were fed an AIN-93G diet, a 1% cholesterol diet (a high cholesterol diet), a high-cholesterol diet containing 4% isoflavone extract of LFS (LFS extract diet), a high-cholesterol diet containing 19.4% ethanol-washed LFS (ethanol-washed LFS diet, isoflavone-poor diet), or a high cholesterol diet containing 23.2% intact LFS (intact LFS diet) for five weeks. The plasma total cholesterol (TC) level was increased in the rats fed the LFS extract diet compared with those fed the high cholesterol diet. The TC level was decreased by the intact LFS and ethanol-washed LFS diets. The cholesterol-lowering effect was stronger in the rats fed the intact LFS diet than those fed the ethanol-washed LFS diet. The plasma triglyceride (TG) level was unchanged in the rats fed the LFS extract diet, but it decreased in rats fed the intact LFS and ethanol-washed LFS diets. Although, compared with the high cholesterol diet, the LFS extract and ethanol-washed LFS diets did not reduce hepatic cholesterol and TG, both levels were remarkably lowered by the intact LFS diet. These results suggest that the improvement in lipid metabolism of rats fed a high-cholesterol diet containing LFS isoflavone aglycones is not due to an independent effect but due to a cooperative effect with soy protein.

## 1. Introduction

Dyslipidemia is one of the risk factors of atherosclerosis [1,2]. Soy foods have been shown to prevent hypercholesterolemia, and soy protein and isoflavones are known as bioactive ingredients of soy foods. Soy protein decreases plasma TC levels [3] by increasing the excretion of fecal bile acid and inhibiting cholesterol absorption in the intestine [4,5]. Several studies have reported that the undigested insoluble fraction of soy protein has a high bile acid-binding activity and stimulates bile acid excretion in the feces [6,7,8]. Soy protein not only lowers plasma TC level but also plasma LDL-C [9,10,11]. Furthermore, β-conglycinin, one of the active components of soy proteins, reduces plasma TG level and body fat mass [12,13,14]. On the other hand, the lipid metabolism-modulation effect of isoflavones has been investigated. The administration of a diet containing 0.03% isoflavone without soy protein affects lipid metabolism in rodents [15,16]. However, an isoflavone-rich soy extract without soy protein or a 1% isoflavone-containing diet without soy protein does not affect lipid metabolism [17,18]. Conversely, an isoflavone-poor soy protein diet has similar lipid metabolism-modulating effects to those exerted by a soy protein diet alone [19,20,21]. There are currently not sufficient data to clarify the independent hypocholesterolemic effect of isoflavones. On the other hand, the lipid metabolism-modulation effect of isoflavones combined with soy protein has already been reported using clinical analysis and meta-analysis [22,23,24,25]. Many researchers have assumed that this modulatory effect of soy foods is achieved by the interaction of soy protein with isoflavones. However, there are no reports on the relationship between the independent effect of isoflavones and the cooperative effect of isoflavones and soy protein on animals.

We focused on fermented soy milk because it exhibits remarkable hypocholesterolemic effects. Soy milk is the simplest of soy foods; however, its taste is generally unfavorable. Thus, we used lactic acid-fermented soy milk (LFS), fermented by lactic acid bacteria of vegetable origin (LFS), to make its taste more palatable. Lactic acid fermentation converts isoflavone glycosides to isoflavone aglycones in soy milk [26] and the conversion enhances their adsorption in the intestine [27]. We have reported the lipid metabolism-modulating effect of LFS in all rats fed an AIN-93G diet [28], a high cholesterol diet [29], a high fat diet [30], and a high fat and high cholesterol diet [31,32]. Additionally, it was found that increasing the ratio of isoflavone aglycones in LFS increases its lipid metabolism-modulating effect [32].

Soy foods reduce serum LDL cholesterol levels in both equol producers and nonproducers, whereas they increase serum HDL-C levels only in equol producers [33]. The isoflavone aglycone-rich LFS may reduce LDL-C and increase HDL-C levels because all rats are equol producers. However, it remains unclear whether soy foods reduce LDL-C via function of isoflavone aglycone alone. It has been reported that soy foods, including soy protein with isoflavone glycones, do not lower plasma LDL-C in either equol producers or nonproducers [34]. The LDL-C-lowering effect of soy foods may depend on the amount of isoflavones and the proportion of their aglycone form. Because most isoflavones in soy foods are in the glycoside form, the independent effect of soy isoflavone aglycones on lipid metabolism-modulating function remains unclear. In the present study, we clarified the role of isoflavone aglycone in the prevention of dyslipidemia using isoflavone aglycone-rich LFS. We examined the independent and cooperative effects of the LFS isoflavone aglycone by conducting two types of experiments. To investigate the independent effect of isoflavone aglycones (Experiment 1), an ethanol extract of LFS without soy protein was administrated to rats fed a high cholesterol diet. In Experiment 2, rats fed a high cholesterol diet were administrated the intact LFS and ethanol-washed LFS diets in order to investigate the effect of soy protein with and without isoflavone aglycones, respectively.

## 2. Materials and Methods

### 2.1. Diets

The LFS was prepared from soy milk by lactic acid fermentation using *Lactobacillus delbrueckii* subsp. *delbrueckii* strain of TUA-4408L (SNC33) for 16 h and immediately freeze-dried for the animal experiments. A fraction including isoflavone and isoflavone aglycone was extracted from LFS using 70% ethanol, evaporated to remove the ethanol, and immediately freeze-dried for animal experimentation. The 70% ethanol extract prepared from LFS was used as the LFS extract. Ethanol-washed LFS was prepared from LFS by washing with 70% ethanol to remove isoflavone and immediately freeze-dried for animal experiments. The composition and energy of LFS are shown in Table 1. The other feed materials were purchased from Clea Japan (Tokyo, Japan), Nacalai Tesque (Kyoto, Japan), and Wako Pure Chemical Industries (Osaka, Japan).

**Table 1:** Composition and energy of several freeze-dried LFS samples.

Component	LFS Extract		Ethanol-Washed LFS		Intact LFS	
	%	Energy (kcal/100 g)	%	Energy (kcal/100 g)	%	Energy (kcal/100 g)
Water	2.9	-	6.7	-	1.6	-
Protein	8.1	32.4	51.8	207.2	43.3	173.2
Fat	1.1	9.9	22.2	199.8	31.9	287.1
Carbohydrate	70.3	281.2	4.2	16.8	4.7	18.8
Dietary fiber	0.0	0.0	11.8	23.6	13.3	26.6
Minerals	17.6	-	3.3	-	5.3	-
Total energy	-	323.5	-	447.4	-	505.7

### 2.2. Animals and Feeding

Fifty-six male Sprague-Dawley rats (seven weeks old) were purchased from Nihon SLC (Hamamatsu, Japan) and individually housed in cages at 23 ± 1 °C and a humidity of 55% ± 7% with a 12 h light-dark cycle. All the rats were acclimated on an AIN-93G diet [35] for one week to stabilize their metabolic conditions before the feeding experiments. In our previous paper [29], an LFS diet containing 10% soy protein as a final concentration enhanced lipid metabolism-modulation effects in rats fed a high cholesterol diet; for that experiment the isoflavone aglycone amount was approximately 30 mg/100 g diet. Thus, in the present study, to investigate the independent effect of isoflavone aglycone, the concentration of isoflavone aglycone was set at approximately 20–30 mg/100 g diet, and an isoflavone fraction extracted with 70% ethanol from LFS (corresponding to 10% soy protein in the diet) was administrated to rats fed a high-cholesterol diet in Experiment 1. In addition, to investigate the cooperative effects of isoflavone aglycone and soy protein in LFS, ethanol-washed fermented LFS and intact LFS were administrated in rats fed a high cholesterol diet in Experiment 2.

#### 2.2.1. Experiment 1

Experiment 1: Effects of isoflavone aglycone extract of LFS on lipid metabolism without soy protein.

Twenty-four rats were assigned to three groups (*n* = 8), which did not exhibit any significant difference in body weight and serum total cholesterol concentration from each other. The control (C) group was fed with the AIN-93G diet. The high cholesterol (H) group was fed with the AIN-93G diet containing 1% cholesterol. The LFS extract (E) group was fed with a diet in which 4% of the high cholesterol diet had been replaced with LFS extract, an isoflavone fraction extracted with 70% ethanol (Table 2). These diets were prepared to contain equal amounts of carbohydrate, protein, fat, and dietary fiber. Thus, the diets were formulated to be isocaloric. Additionally, the isoflavone content in the LFS extract diet was 20.6 mg/100 g diet, corresponding to approximately 0.02% of the diet (Table 3). The isoflavone aglycone content was 12.3 mg/20.6 mg total isoflavone (Table 3). The rats in each group were fed for five weeks and provided with permitted *ad libitum* access to food and water. The food intake and body weight were measured as described in our previous paper [28]. Analyses of the blood and liver were carried out as described in the previous paper [28]. Feces were collected for two days in the final week.

#### 2.2.2. Experiment 2

Experiment 2: Effects of ethanol-washed LFS without soy protein and intact LFS on lipid metabolism with soy protein.

Thirty-two rats were assigned to four groups (*n* = 8), which did not exhibit any significant difference in body weight and serum total cholesterol concentration from each other. The control (C) group was fed with the AIN-93G diet. The high cholesterol (H) group was fed with the AIN-93G diet containing 1% cholesterol. LFS washed with 70% ethanol was prepared as soy protein including low isoflavone. The ethanol-washed LFS (W) group was fed with a diet in which 19.4% of the high cholesterol diet had been replaced with dried ethanol-washed LFS and contained 10% soy protein as final concentration, and the LFS (F) group was fed with a diet in which 23.2% of the high cholesterol diet had been replaced with LFS and contained 10% soy protein as a final concentration (Table 2). These diets were prepared to contain equal amounts of carbohydrate, protein, fat and dietary fiber. Thus, the diets were formulated to be isocaloric. Additionally, the isoflavone content of the intact LFS diet was 32.4 mg/100 g diet, corresponding to approximately 0.03% of the diet. The isoflavone aglycone content was 28.3 mg/32.4 mg total isoflavone. On the other hand, ethanol-washed LFS contained 1.1 mg isoflavone aglycone /100 g diet, corresponding to only 0.001% of the diet (Table 3). The isoflavone aglycone content was 0.3 mg/1.1 mg total isoflavone. For feeding procedures, the same method as described in Experiment 1 was used.

These animal experiments were performed according to the guidelines of the Animal Use Committee of Mukogawa Women’s University, and the experiments were approved by the Committee for the Care and Use of Laboratory Animals at Mukogawa Women’s University on 1 May 2013 (code: P2011013).

**Table 2:** Composition of the experimental diets.

Ingredient	Diet Group				
	C: AIN-93G *	H	E	W	F
Casein ^1^ (%)	20.0	20.0	19.6	8.4	8.4
Cornstarch ^1^ (%)	39.8	38.8	36.3	38.0	38.0
Dextrinized cornstarch ^1^ (%)	13.2	13.2	12.1	12.7	12.6
Sucrose ^1^ (%)	10.0	10.0	10.0	10.0	10.0
Soybean oil ^1^ (%)	7.0	7.0	7.0	2.9	0.0
Cellulose ^1^ (%)	5.0	5.0	5.0	2.6	1.8
Mineral mix (AIN-93G-MX) ^1^ (%)	3.5	3.5	3.5	3.5	3.5
Vitamin mix (AIN-93-VX) ^1^ (%)	1.0	1.0	1.0	1.0	1.0
l-Cystine ^2^ (%)	0.3	0.3	0.3	0.3	0.3
Choline bitartrate ^3^ (%)	0.25	0.25	0.25	0.25	0.25
*tert*-Butylhydroquinone ^2^ (%)	0.0014	0.0014	0.0014	0.0014	0.0014
Cholesterol ^2^ (%)	0.0	1.0	1.0	1.0	1.0
Fermented soy milk extract ^4^	0.0	0.0	4.0	0.0	0.0
Ethanol-washed fermented soy milk ^4^	0.0	0.0	0.0	19.4	0.0
Fermented soy milk ^4^ (%)	0.0	0.0	0.0	0.0	23.2
Total (%)	100.0	100.0	100.0	100.0	100.0
Energy (kcal/100 g)	372.2	368.7	367.4	367.0	369.7

C, control; H, 1% cholesterol diet; E, 4% of the high cholesterol diet was replaced with LFS extract; W, 19.4% of the high cholesterol diet was replaced with dried ethanol-washed LFS to include 10% soy protein as a final concentration; F, 23.2% of the high cholesterol diet was replaced with LFS to include 10% soy protein as a final concentration; ^1^ Clea Japan, Osaka, Japan; ^2^ Wako Pure Chemical Industries, Osaka, Japan; ^3^ Nacalai Tesque, Kyoto, Japan; ^4^ Marusan-Ai, Okazaki, Japan; * AIN-93G diet [35].

**Table 3:** Isoflavone composition of the experimental diets (mg/100g diet).

Component	Diet Group		
	E	W	F
Daidzin	0.5	0.0	0.2
Malonyl daidzin	2.1	0.2	1.2
Acetyl daidzin	0.4	0.0	0.3
Daidzein	4.4	0.1	11.2
Genistin	0.8	0.0	0.2
Malonyl genistin	4.4	0.4	2.0
Acetlyl genistin	0.0	0.0	0.0
Genistein	7.7	0.2	16.7
Glycitin	0.1	0.0	0.2
Malonyl glycitin	0.0	0.1	0.0
Acetly glycitin	0.0	0.0	0.0
Glycitein	0.2	0.0	0.3
Total daizein (glycine + aglycone)	7.4	0.4	12.9
Total genistein (glycine + aglycone)	12.9	0.6	18.9
Total glycitein (glycine + aglycone)	0.3	0.1	0.5
Total isoflavone(glycine + aglycone)	20.6	1.1	32.4
Total isoflavone aglycone	12.3	0.3	28.3
Aglycone ratio (%)	59.6	30.9	87.3

E, 4% of the high cholesterol diet was replaced with LFS extract; W, 19.4% of the high cholesterol diet was replaced with dried ethanol-washed LFS to include 10% soy protein as a final concentration; F, 23.2% of the high cholesterol diet was replaced with LFS to include 10% soy protein as a final concentration.

### 2.3. Measurement of the Plasma, Hepatic, and Fecal Metabolic Parameters

Plasma TC, HDL-C and TG were enzymatically measured by using commercial kits (Cholesterol E-test, HDL-cholesterol E-test and Triglyceride E-test, Wako Pure Chemical Industries, Osaka, Japan). The non-HDL-C concentration was calculated as (non-HDL-C) = (TC) − (HDL-C). The hepatic and fecal lipids were extracted by the ordinary method of Folch *et al.* [36]. The hepatic cholesterol, TG, and fecal cholesterol concentrations were enzymatically determined by using commercial kits (Cholesterol E-test and Triglyceride E-test, Wako Pure Chemical Industries, Osaka, Japan). Fecal bile acids were extracted with hot ethanol (70 °C, 60 min) from freeze-dried feces. The extracts were enzymatically determined by using commercial kits (Total bile acid test, Wako Pure Chemical Industries, Osaka, Japan).

### 2.4. X-Ray Computed Tomography (CT) Analysis

All the rats were subjected to X-ray computed tomography (CT) analysis using LaTheta LCT-200 (Aloka, Tokyo, Japan) under anesthesia at the initial and end points of the feeding period. Measurement of CT was carried out as described in our previous paper [29]. Total, visceral, and subcutaneous fats were distinguished by CT value and evaluated quantitatively. The difference of each fat mass between the initial and end points of the feeding period was measured.

### 2.5. Real-Time Reverse Transcription-Polymerase Chain Reaction (RT-PCR)

The expression of mRNA was quantitatively measured by real-time RT-PCR, using the model 7500 (Applied Biosystems, Foster City, CA, USA) and related reagent kits according to the manufacturer’s protocol. The extraction of total RNA and synthesis of complementary DNA (cDNA) were carried out as described in the previous paper [29]. The following TaqMan gene expression assays were conducted using Rn01495769_ml for Srebf1 (mRNA of SREBP-1), Rn00569117_ml for Fasn (mRNA of FAS), Rn00581185_ml for Nr1h3 (mRNA of LXRα), Rn01502638_ml for Srebf2 (mRNA of SREBP-2), Rn00580702_m1 for Cpt1a (mRNA of CPT1a), Rn00572658_m1 for Nrlh4 (mRNA of FXR), Rn00598438_m1 for Ldlr (mRNA of LDLR), Rn00565598_m1 for Hmgcr (mRNA of HMG-CoA R), and Rn00564065_ml for Cyp7a1 (mRNA of CYP7a1) (Applied Biosystems) as the PCR primer set for the real-time PCR. Rn99999916_s1 for GAPDH was used as an endogenous control. Real-time PCR was performed with the TaqMan Universal PCR master mix (Applied Biosystems). Data were normalized to GAPDH RNA expression, and the fold was presented as a ratio to the C group.

### 2.6. Measurement of Isoflavone in the Diet and Plasma

For the measurement of isoflavone, plasma was extracted with 70% ethanol at room temperature for 24 h and centrifuged at 10,000× *g* for 30 min at room temperature. Plasma had been previously deconjugated by β-glucuronidase (Sigma-Aldorich, Tokyo, Japan). The supernatant was collected and filtered through a cellulose acetate membrane (Tosoh, Japan). Isoflavones in diet and plasma were analyzed by HPLC (Tosoh, Japan) equipped with an ODS-80Ts column. HPLC was performed by gradient elution of acetonitrile from 15% to 35% using solvents of sodium acetate (pH 4.8)-methanol (80:20) and sodium acetate (pH 4.8)-methanol-acetonitrile (40:20:20).

### 2.7. Statistical Analysis

The results are presented as the mean ± standard error, and were analyzed by Tukey’s multiple-comparison test at *p* < 0.05. The statistical analyses were performed with SPSS 12.0 J for Windows.

## 3. Results

### 3.1. Body Weight, Food Intake, Food Efficiency, Total Energy Intake, and Tissue Weights in Experiment 1

No significant differences in final body weight, food intake, food efficiency, and total energy intake were found between the three groups (Table 4).

The weights of the livers in the H and E groups were significantly higher than that in the C group. The weight of cecum in the E group was significantly increased over that in the C and H groups.

**Table 4:** Initial and final body weights, food intake, food efficiency, total energy intake, and tissue weights of rats fed on the experimental diets for five weeks in Experiment 1.

Body Weight and Food Efficiency	C	H	E
Initial body weight (g)	253.7 ± 4.1 ^a^	257.2 ± 3.3 ^a^	256.6 ± 3.0 ^a^
Final body weight (g)	429.3 ± 10.1 ^a^	440.6 ± 10.5 ^a^	427.6 ± 10.4 ^a^
Food intake (g/day)	20.3 ± 0.6 ^a^	21.1 ± 0.7 ^a^	19.7 ± 0.6 ^a^
Food efficiency (g b.w. gain/g diet)	0.23 ± 0.01 ^a^	0.23 ± 0.02 ^a^	0.23 ± 0.01 ^a^
Total energy uptake (kcal)	2896.5 ± 75.7 ^a^	2992.9 ± 103.3 ^a^	2758.8 ± 74.4 ^a^
*Tissue weight (% b.w.)*			
Liver	3.4 ± 0.1 ^a^	4.3 ± 0.1 ^b^	4.6 ± 0.1 ^b^
Cecum	0.8 ± 0.0 ^a^	0.8 ± 0.1 ^a^	1.1 ± 0.1 ^b^

Each value is the mean ± SE for eight rats. ^a, b^ Means not sharing a common superscript differ significantly according to Tukey’s multiple-comparison test (*p* < 0.05).

### 3.2. Hepatic Lipid Profiles in Experiment 1

**Figure 1:**
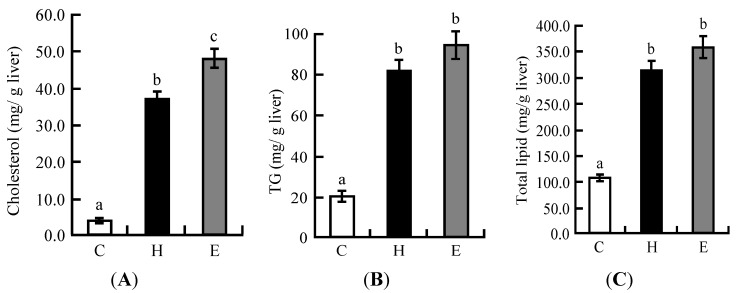
Liver parameters of rats fed on the experimental diets for five weeks in Experiment 1: (**A**) hepatic cholesterol; (**B**) hepatic TG; (**C**) hepatic total lipid. Each value is the mean ± SE for eight rats. ^a, b, c^ Means not sharing a common superscript differ significantly according to Tukey’s multiple-comparison test (*p* < 0.05).

The hepatic cholesterol level was significantly higher in the H and E groups than in the C group (Figure 1A). In particular, the hepatic cholesterol level was significantly higher in the E group than in the H group. Hepatic TG and total lipid levels in the H and E groups were higher than those in the C group (Figure 1B,C).

### 3.3. Plasma Lipid Profiles in Experiment 1

The H group, fed a high cholesterol diet, showed a higher level of plasma TC than the C group, but the TC level in the E group was significantly increased compared with that in the H group (Table 5). After the five weeks, the plasma TG levels were unchanged in all three groups (Table 5).

### 3.4. Real-Time PCR Analysis in Experiment 1

**Figure 2:**
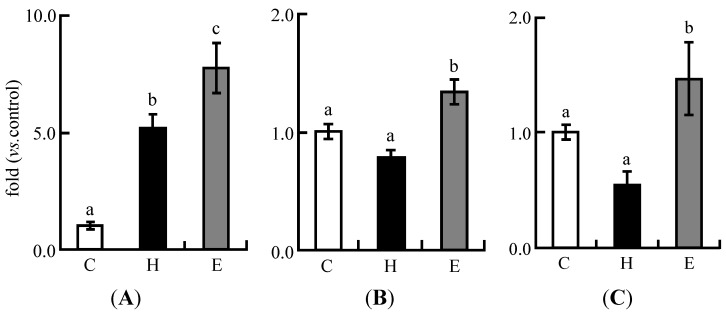

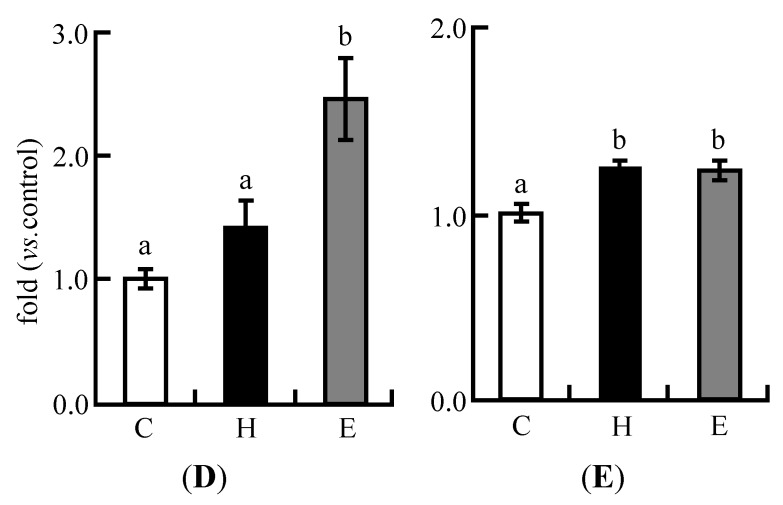
Expression of lipid metabolism-related genes in the livers of rats fed experimental diets for five weeks in Experiment 1. The expression of mRNA was quantitatively measured by real-time RT-PCR. (**A**) CYP7a1; (**B**) SREBP-2; (**C**) FAS; (**D**) SREBP-1; (**E**) LXRα. Each value is the mean ± SE for eight rats. The data were normalized to GAPDH RNA expression and are presented as a ratio to the C value. ^a, b, c^ Means not sharing a common superscript differ significantly according to Tukey’s multiple-comparison test (*p* < 0.05).

During cholesterol metabolism, the expression of CYP7a1, the rate-limiting enzyme during the formation of bile acid from cholesterol, was up-regulated in the H group, which was fed a high cholesterol diet. The expression of this enzyme in the E group was significantly up-regulated compared to the C and H groups (Figure 2A). The expression of SREBP-2 was significantly increased in the E group compared with the C and H groups (Figure 2B). The expression of fatty acid synthesis-related enzyme was also increased in the E groups compared with the C and H groups (Figure 2C). The expression of SREBP-1, which controls the fatty acid synthesis-related pathway, was also up-regulated in the E group compared with the H and C groups (Figure 2D). LXR α, the nuclear receptor controlling the expression of SREBP-1 and SREBP-2, was up-regulated in the H and E groups compared with the C group (Figure 2E).

**Table 5:** Plasma parameters of rats fed on the experimental diets for five weeks in Experiment 1.

Lipid Concentration	C	H	E
*Total cholesterol (mmol/L)*			
0W	2.24 ± 0.10 ^a^	2.25 ± 0.13 ^a^	2.24 ± 0.07 ^a^
1W	1.60 ± 0.06 ^a^	2.22 ± 0.06 ^b^	2.75 ± 0.07 ^c^
2W	1.63 ± 0.11 ^a^	2.19 ± 0.03 ^b^	2.51 ± 0.16 ^b^
3W	1.74 ± 0.11 ^a^	2.24 ± 0.05 ^b^	2.70 ± 0.14 ^c^
4W	1.69 ± 0.11 ^a^	1.98 ± 0.05 ^ab^	2.42 ± 0.19 ^b^
5W	1.86 ± 0.11 ^a^	2.19 ± 0.06 ^a^	2.75 ± 0.16 ^b^
*Triglyceride (mmol/L)*			
0W	0.72 ± 0.09 ^a^	0.71 ± 0.08 ^a^	0.73 ± 0.07 ^a^
1W	1.23 ± 0.15 ^a^	1.44 ± 0.12 ^a^	1.58 ± 0.14 ^a^
2W	1.53 ± 0.31 ^a^	1.73 ± 0.20 ^a^	1.26 ± 0.14 ^a^
3W	1.41 ± 0.21 ^a^	1.59 ± 0.15 ^a^	1.36 ± 0.20 ^a^
4W	1.48 ± 0.20 ^a^	1.44 ± 0.15 ^a^	1.32 ± 0.16 ^a^
5W	1.37 ± 0.20 ^a^	1.19 ± 0.11^a^	1.21 ± 0.22 ^a^

Each value is the mean ± SE for 8 rats. ^a, b, c^ Means not sharing a common superscript differ significantly according to Tukey’s multiple-comparison test (*p* < 0.05).

### 3.5. Body Weight, Food Intake, Food Efficiency, Total Energy Intake, and Tissue Weights in Experiment 2

No significant differences were found in final body weight, food intake, food efficiency, and total energy intake between the four groups (Table 6).

The weights of livers were higher in the H and W groups than the C group, whereas the weights of the livers in the F group were significantly lower than in the H and W groups. The weight of cecum in the F group was significantly increased compared with that in the other groups.

**Table 6:** Initial and final body weights, food intake, food efficiency, total energy intake, and tissue weights of rats fed on the experimental diets for five weeks in Experiment 2.

Body Weight and Food Efficiency	C	H	W	F
Initial body weight (g)	271.7 ± 1.7 ^a^	273.1 ± 3.4 ^a^	269.4 ± 3.3 ^a^	271.7 ± 1.4 ^a^
Final body weight (g)	458.0 ± 10.0 ^a^	480.6 ± 12.8 ^a^	468.1 ± 10.2 ^a^	457.0 ± 4.2 ^a^
Food intake (g/day)	20.8 ± 0.5 ^a^	20.8 ± 0.5 ^a^	20.6 ± 0.4 ^a^	21.2 ± 0.4 ^a^
Food efficiency (g b.w. gain/g diet)	0.24 ± 0.01 ^a^	0.26 ± 0.02 ^a^	0.26 ± 0.01 ^a^	0.23 ± 0.01^a^
Total energy uptake (kcal)	2932.6 ± 68.2 ^a^	2905.0 ± 67.6 ^a^	2835.5 ± 59.2 ^a^	2948.9 ± 64.8 ^a^
*Tissue weight (% b.w.)*				
Liver	3.8 ± 0.1 ^a^	4.9 ± 0.1 ^b^	4.5 ± 0.1 ^b^	4.1 ± 0.1 ^a^
Cecum	1.0 ± 0.0 ^a^	1.1 ± 0.1 ^a^	1.0 ± 0.0 ^a^	1.3 ± 0.1 ^b^

Each value is the mean ± SE for 8 rats. ^a, b^ Means not sharing a common superscript differ significantly according to Tukey’s multiple-comparison test (*p* < 0.05).

### 3.6. Visceral Fat and Subcutaneous Fat Masses

**Figure 3:**
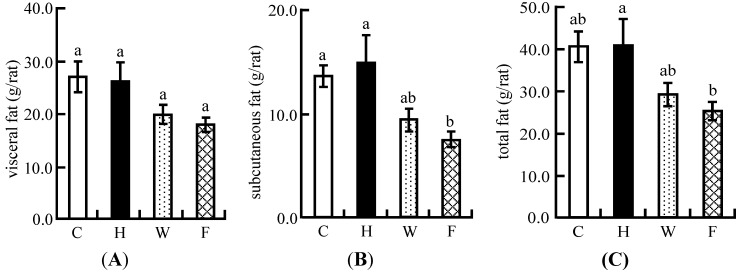
Comparison of increased amount of visceral and subcutaneous fat masses in three groups in Experiment 2: (**A**) visceral fat; (**B**) subcutaneous fat; (**C**) total fat. The difference of each fat mass between the initial and end points of the feeding period was measured. These fat mass regions were estimated by X-ray CT scan. Each value is the mean ± SE of eight rats. ^a, b^ Means not sharing a common superscript differ significantly according to Tukey’s multiple-comparison test (*p* < 0.05).

The increase in the amount of visceral fat mass was not significantly decreased in the W and F groups compared with that of the H group (Figure 3A). On the other hand, in the F group, the increase in the amount of subcutaneous fat and total fat was significantly lower compared with the H group. (Figure 3B,C).

### 3.7. Hepatic Lipid Profile

**Figure 4:**
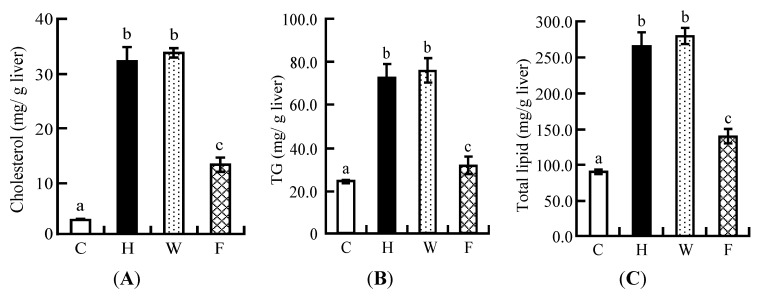
Liver parameters of rats fed on the experimental diets for five weeks in Experiment 2: (**A**) hepatic cholesterol; (**B**) hepatic TG; (**C**) hepatic total lipid. Each value is the mean ± SE for eight rats. ^a, b, c^ Means not sharing a common superscript differ significantly according to Tukey’s multiple-comparison test (*p* < 0.05).

Compared with the C group, hepatic lipid, cholesterol, and TG levels were higher in the H group but significantly lower in the F group. However, these levels were not affected in the W group compared with the H group (Figure 4A–C).

### 3.8. Plasma Lipid Profiles

The H group, fed a high cholesterol diet, showed a higher level of plasma TC compared with the C group; the levels in the W group were significantly decreased, reaching those observed in the C group. Moreover, the TC level in the F group was significantly decreased compared with that in the W group from the third to the fifth week of the experiment (Table 7). The plasma non-HDL-C level also increased in the H group, but the level was significantly lower in the F group than in the W group from the third to the fifth week. The plasma TG level was decreased in the W and F groups compared with the H group. There was no significant difference in TG levels between the W and F groups.

**Table 7:** Plasma parameters of rats fed on the experimental diets for five weeks in Experiment 2.

Lipid Concentration	C	H	W	F
*Total cholesterol (mmol/L)*				
0W	2.03 ± 0.09 ^a^	1.97 ± 0.08 ^a^	2.03 ± 0.13 ^a^	1.99 ± 0.13 ^a^
1W	1.88 ± 0.08 ^a^	2.33 ± 0.09 ^b^	1.80 ± 0.10 ^ac^	1.48 ± 0.07 ^c^
2W	1.68 ± 0.09 ^a^	2.25 ± 0.12 ^b^	1.65 ± 0.07 ^a^	1.34 ± 0.05 ^a^
3W	1.80 ± 0.06 ^ac^	2.35 ± 0.13 ^b^	1.86 ± 0.08 ^a^	1.46 ± 0.07 ^c^
4W	2.03 ± 0.07 ^a^	2.55 ± 0.15 ^b^	2.02 ± 0.09 ^a^	1.48 ± 0.07 ^c^
5W	2.15 ± 0.10 ^a^	2.73 ± 0.18 ^b^	1.97 ± 0.07 ^a^	1.50 ± 0.08 ^c^
*non-HDL-C (mmol/L)*				
0W	0.49 ± 0.06 ^a^	0.53 ± 0.04 ^a^	0.56 ± 0.06 ^a^	0.52 ± 0.06 ^a^
1W	0.54 ± 0.04 ^a^	1.43 ± 0.10 ^b^	1.09 ± 0.10 ^c^	0.80 ± 0.05 ^ac^
2W	0.50 ± 0.05 ^a^	1.50 ± 0.14 ^b^	1.05 ± 0.07 ^c^	0.77 ± 0.05 ^ac^
3W	0.44 ± 0.03 ^a^	1.54 ± 0.15 ^b^	1.14 ± 0.08 ^c^	0.69 ± 0.05 ^a^
4W	0.62 ± 0.06 ^a^	1.79 ± 0.15 ^b^	1.42 ± 0.08 ^c^	0.89 ± 0.05 ^a^
5W	0.49 ± 0.03 ^a^	1.79 ± 0.17 ^b^	1.21 ± 0.08 ^c^	0.75 ± 0.07 ^a^
*Triglyceride (mmol/L)*				
0W	1.05 ± 0.19 ^a^	1.06 ± 0.06 ^a^	1.07 ± 0.15 ^a^	1.08 ± 0.09 ^a^
1W	1.12 ± 0.09 ^a^	1.88 ± 0.12 ^b^	1.51 ± 0.26 ^ab^	1.42 ± 0.12 ^ab^
2W	1.36 ± 0.09 ^a^	1.99 ± 0.18 ^b^	1.29 ± 0.11 ^a^	1.38 ± 0.16 ^a^
3W	1.66 ± 0.11 ^ab^	2.18 ± 0.17 ^b^	1.51 ± 0.20 ^a^	1.34 ± 0.09 ^a^
4W	1.63 ± 0.15 ^a^	1.97 ± 0.23 ^a^	1.51 ± 0.11 ^a^	1.43 ± 0.12 ^a^
5W	1.40 ± 0.09 ^a^	1.89 ± 0.16 ^b^	1.43 ± 0.11 ^ab^	1.38 ± 0.10 ^a^

Each value is the mean ± SE for eight rats. ^a, b, c^ Means not sharing a common superscript differ significantly according to Tukey’s multiple-comparison test (*p* < 0.05).

### 3.9. Real-Time PCR Analysis

**Figure 5:**
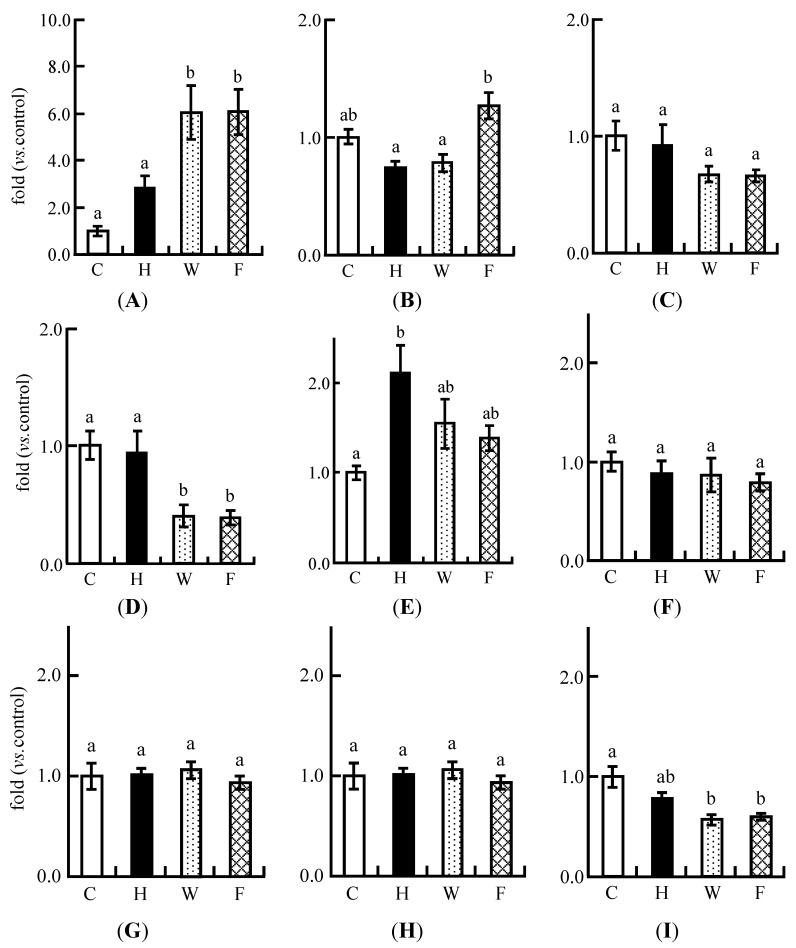
Expression of lipid metabolism-related genes in the livers of rats fed experimental diets for five weeks in Experiment 2. The expression of mRNA was quantitatively measured by real-time RT-PCR. (**A**) CYP7a1; (**B**) SREBP-2; (**C**) HMGCR; (**D**) FAS; (**E**) SREBP-1; (**F**) CPT1a; (**G**) LXRα; (**H**) FXR; (**I**) LDLR. Each value is the mean ± SE for eight rats. The data were normalized to GAPDH RNA expression and are presented as a ratio to the C value. ^a, b^ Means not sharing a common superscript differ significantly according to Tukey’s multiple-comparison test (*p* < 0.05).

The expression of CYP7a1 was up-regulated in the H group, and was significantly up-regulated in the W and F groups compared with the C and H groups (Figure 5A). There was no significant difference with regard to this between the W and F groups. The expression of SREBP-2 was significantly increased in the F group compared with the H and W groups (Figure 5B). However, the expression of the HMGCR gene, a coding rate-limiting enzyme, tended to decrease in the W and F groups compared with the C and H groups (Figure 5C).

On the other hand, the expression of FAS was decreased in the W and F groups compared with the C and H groups (Figure 5D). The expression of SREBP-1 was up-regulated in the H group compared with the C group, whereas there was no difference with regard to this between the W and F groups (Figure 5E). The expression of CPT-1a, a beta oxidation-related enzyme, did not differ between the four groups (Figure 5F). LXR α was up-regulated in the H and W groups compared with the C group (Figure 5G). The expression of the FXR gene was not affected in any of the groups (Figure 5H). The expression of LDLR was decreased in the W and F groups compared with the C group (Figure 5I).

## 4. Discussion

We conducted two experiments. In Experiment 1, we investigated the effects of isoflavone aglycones in LFS alone on the lipid metabolism of rats fed a high cholesterol diet. In Experiment 2, we examined the cooperative effect of isoflavone aglycones and soy protein on the lipid metabolism of rats fed a high cholesterol diet by comparing the effect of administering intact LFS and ethanol-washed LFS diets.

In Experiment 1, the weights of the livers of rats in the high cholesterol diet and LFS extract diet groups was clearly increased. The hepatic lipid, cholesterol, and TG levels were significantly higher in the high cholesterol and LFS extract diet groups compared with the control group. In particular, the cholesterol level was significantly higher in the LFS extract diet group compared with the high cholesterol diet group. In addition, the plasma TC level significantly increased in the LFS extract diet group compared with the high cholesterol diet group, whereas plasma TG levels did not differ between the three groups.

In our previous studies [28,29,30,31,32], we found that the gene expression of hepatic CYP7a1, a rate-limiting enzyme during the formation of bile acid from cholesterol [37], controlled by LXRα [38], increased in the LFS diet group fed an AIN-93G diet [28], a high cholesterol diet [29], a high fat diet [30], and a high fat and high cholesterol diet [31,32]. Notably, we have demonstrated that LFS with a higher proportion of isoflavone aglycones enhances the gene expression of CYP7a1 compared with LFS, with a lower proportion of isoflavone aglycones [32]. In the present study, the expression of CYP7a1 was higher in the LFS extract diet group than in the high cholesterol diet group (Figure 2A). On the other hand, the gene expression of SREBP-2, a cholesterol synthesis-accelerating factor [39], was increased in the LFS extract diet group compared with the high cholesterol diet group (Figure 2B). Therefore, we assumed that, in the LFS extract diet group, the hepatic cholesterol and plasma TC levels were increased because of accelerated cholesterol synthesis rather than cholesterol catabolism in the liver. The expression of FAS was higher in the LFS extract diet group than in the high cholesterol group (Figure 2C). The expression of SREBP-1, the gene up-stream of FAS [40], was also increased in the LFS extract diet group compared with the high cholesterol diet group (Figure 2D). The expression of SREBP-1 and FAS genes is controlled by LXRα [41]. The expression of the LXRα gene was higher in the high cholesterol and the LFS extract diet groups compared with the control group, but the difference was not significant between the high cholesterol and LFS extract diet groups (Figure 2E). Thus, the expression of LXRα was stimulated by the high cholesterol diet and the LFS extract diet. We can assume that the hepatic TG levels in the LFS extract group were increased by promoting fatty acid synthesis-related gene expression.

These results indicated that the LFS extract promoted both cholesterol and fatty acid synthesis in the rats fed a high cholesterol diet and increased hepatic and plasma lipid levels. Mullen *et al.* [42] have reported the up-regulation of SREBP-2 caused by isoflavones. We observed that the expression of the fatty acid synthesis gene was accelerated in Hep G2 cells after the addition of isoflavones (unpublished data). Thus, it was found that isoflavone aglycones in LFS do not independently improve lipid metabolism in rats fed a high cholesterol diet but conversely accelerate dyslipidemia.

We also investigated the cooperative effects of isoflavone aglycones and soy protein on lipid metabolism in rats fed a high cholesterol diet by comparing the effects of administering intact LFS and ethanol-washed LFS diets in Experiment 2.

The body fat mass was significantly decreased in the intact LFS diet group compared with the high cholesterol diet group, whereas there was no significant decrease in the ethanol-washed LFS diet group (Figure 3). The reduction of body fat mass appeared to be associated not only with soy proteins but also with isoflavone aglycones. The hepatic lipid, cholesterol, and TG levels were lower in the intact LFS diet group compared with the high cholesterol diet group, but the ethanol-washed LFS diet did not have such effects (Figure 4). We found that the reduction of hepatic lipid, cholesterol, and TG levels caused by the ingestion of the intact LFS diet decreased the weight of the liver (Table 6). These results indicated that the decrease in liver weight depended on the interaction of isoflavone aglycones and soy protein.

Plasma TC and non-HDL-C levels decreased with the administration of both LFS diets (Table 7). However, the lowering effect was greater in the intact LFS diet group than in the ethanol-washed LFS diet group. It has been shown that soy protein reduces plasma LDL-C levels [9,10,11]. Soy food consumption reduces serum LDL-C levels in both equol producers and nonproducers, whereas it Modificationincreases serum HDL-C levels only in equol producers [34]. In the present study, LDL-C level was reduced in the intact LFS diet group, suggesting that this effect was stimulated by isoflavone aglycones. However HDL-C level were not increased by isoflavone aglycones. Additionally, plasma TG levels were decreased by both LFS diets regardless of the isoflavone aglycone content (Table 7). These results indicated that the reduction in hepatic cholesterol, hepatic TG, plasma TC, and plasma LDL-C levels depended on the amount of isoflavone aglycones ingested along with soy protein. However, isoflavone aglycones did not affect plasma TG and HDL-C levels. Fukui *et al.* [43] have observed that isoflavone-free soy protein reduces plasma cholesterol levels in rats and that this effect was not achieved when this isoflavone was added. Because their study used isoflavone glycoside, the potential role of isoflavone aglycones has not been excluded. The results of the present study suggested that isoflavone aglycones promoted the cholesterol-lowering effect of soy protein.

It was not clear whether the phenomenon of lowering hepatic and plasma lipid levels depends on the excretion of cholesterol and bile acid in feces or not. Therefore, we examined these substances in the feces of rats fed different diets for five weeks. The amount of cholesterol excreted in feces was 4.4 mg/day in rats fed a control (AIN-93G) diet. The amount of excreted cholesterol increased in the high cholesterol diet, ethanol-washed LFS diet, and intact LFS diet groups to 32.6, 27.5, and 27.8 mg/day, respectively. The amount of bile acid excreted was 20.7 mg/day in rats fed a control diet, and no significant differences were found between the other diet groups (100.2, 96.3, and 81.6 mg/day for high cholesterol, ethanol-washed LFS, and intact LFS diet groups, respectively). Thus, the decrease in hepatic lipid and plasma TC levels could not be explained by changes in the amounts of cholesterol and bile acid that were excreted. Therefore, we investigated the lipid metabolism related-gene expression in the liver.

We predicted that the expression of CYP7a1 would be up-regulated in the intact LFS diet group compared with the ethanol-washed LFS diet group. However, regardless of the amount of isoflavone aglycones in LFS, the expression of CYP7a1 remained the same in both groups (Figure 5A). The gene expression of the SREBP-2 gene was higher in the intact LFS group compared with that of the high cholesterol and ethanol-washed LFS diet groups (Figure 5B). A decrease in intracellular levels of cholesterol appears to enhance SREBP-2 gene expression [44]. We assumed that from the early to middle feeding period, the gene expression of the CYP7a1 gene and excretion of bile acid in feces would be promoted in the intact LFS diet group compared with the ethanol-washed LFS diet group. However, it seems that the expression of CYP7a1 was decreased and that of SREBP-2 was increased in the intact LFS diet group after five weeks in order to maintain cholesterol homeostasis. As a result, no significant difference in CYP7a1 expression levels between these two groups was found after five weeks. The expression of LXR, which controls CYP7a1, did not change in the high cholesterol, ethanol-washed LFS, or intact LFS diet groups (Figure 5G). The expression of FXR, which inhibits CYP7a1 [38], was also not affected by consumption of LFS (Figure 5H). On the other hand, the expression of the HMGCR gene, which codes for a rate-limiting enzyme in the synthesis of cholesterol [45], was slightly decreased in the ethanol-washed LFS and intact LFS diet groups (Figure 5C). It appears that a slight decrease in HMGCR expression and a significant increase in CYP7a1 expression reduced hepatic cholesterol levels in the ethanol-washed LFS and intact LFS diet groups. LDLR is up-regulated by soy protein and isoflavones [15,46]. Increasing LDLR protein levels promotes the endocytosis of cholesterol from the blood to the liver. Because the excessive hepatic cholesterol provided by the high cholesterol diet needs to induce its own secretion in the blood, we assumed that the expression of LDLR would be decreased in the ethanol-washed LFS and intact LFS diet groups (Figure 5I). The results of the present study suggested that the decrease in plasma TC and non-HDL-C levels are caused by the inhibition of cholesterol synthesis and promotion of cholesterol excretion. Thus, it is likely that hepatic intracellular cholesterol levels are decreased by the acceleration of CYP7a1 expression and reduction of HMGCR and LDLR expressions in rats fed an intact LFS diet.

The gene expressions of FAS and SREBP-1 were higher in the LFS extract diet group than in the high cholesterol diet group in Experiment 1 (Figure 2C,D). In contrast, the gene expression of FAS was down-regulated in the ethanol-washed LFS and intact LFS diet groups compared with the high cholesterol diet group (Figure 5D). SREBP-1 was also slightly down-regulated in the ethanol-washed LFS and intact LFS diet groups compared with the high cholesterol diet group (Figure 5E). The expression of the LXRα gene did not change in the ethanol-washed LFS and intact LFS diet groups. These results suggested that the down-regulation of FAS and SREBP-1 expression caused by isoflavone aglycones in LFS is not due to an independent effect, but due to a cooperative effect with soy protein. On the other hand, the expression of CPT-1a in the β-oxidation pathway of fatty acids [47] was not affected by either LFS diet (Figure 5F). There was a small difference in the hepatic gene expression levels of fatty acid synthesis between the ethanol-washed LFS and intact LFS diet groups. In our previous paper [27], we observed that fatty acid synthesis-related gene expression is more markedly down-regulated after one week than after five weeks. Thus, in this study, we assumed that the decrease in hepatic TG levels was caused by the inhibition of fatty acid synthesis-related gene expression in the early feeding period in the intact LFS diet group. Although all liver parameters of the intact LFS diet group were significantly lower than in the ethanol-washed diet group, the differences in the expression levels of lipid metabolism-related genes between these groups were small because the expression change had already decreased. It is possible that a prolonged feeding time reduces the effect on the expression of lipid metabolism genes as Kanamoto *et al.* [48] have suggested in a study using soybean protein.

Because the peptide content of soy milk was not altered by lactic fermentation, it appears that there are no newly produced peptides from soy milk protein. We have examined the effect of gene expression by peptides prepared from soy milk using HepG2 (unpublished data), and found that peptides from fermented soy milk do not affect cholesterol catabolism.

The LFS extract without soy protein promoted hepatic cholesterol and fatty acid synthesis in rats fed a high cholesterol diet. Conversely, the intact LFS diet suppressed cholesterol and fatty acid synthesis compared with the ethanol-washed LFS diet. We found that the isoflavone aglycones of LFS alone do not improve lipid metabolism in rats fed a high cholesterol diet, but they improved lipid metabolism when combined with soy protein.

## 5. Conclusions

We suggested that isoflavones and protein in LFS combine to prevent dyslipidemia in rats fed a high cholesterol diet. Therefore, the consumption of LFS rich in isoflavone aglycones and soy protein might help to prevent dyslipidemia.

## Abbreviations

LFS: lactic acid-fermented soymilk
TC: total cholesterol
TG: triglyceride
LDL-C: low density lipoprotein cholesterol
HDL-C: high density lipoprotein cholesterol
CYP7a1: cytochrome p450 family 7 subfamily a polypeptide 1
FAS: fatty acid synthase
GAPDH: glyceraldehyde-3-phosphate dehydrogenase
LXRα: liver X receptor alpha
SREBP-1: sterol regulatory element binding protein 1
SREBP-2: sterol regulatory element binding protein 2
HMGCR: 3-hydroxy-3-methylglutaryl-CoA reductase
CPT-1: carnitine palmitoyltransferase 1
FXR: farnesoid X receptor
LDLR: low-density lipoprotein receptor
